# Is Fluid Overload More Important than Diabetes in Renal Progression in Late Chronic Kidney Disease?

**DOI:** 10.1371/journal.pone.0082566

**Published:** 2013-12-09

**Authors:** Yi-Chun Tsai, Jer-Chia Tsai, Yi-Wen Chiu, Hung-Tien Kuo, Szu-Chia Chen, Shang-Jyh Hwang, Tzu-Hui Chen, Mei-Chuan Kuo, Hung-Chun Chen

**Affiliations:** 1 Division of Nephrology, Kaohsiung Medical University Hospital, Kaohsiung, Taiwan; 2 Department of Nursing, Kaohsiung Medical University Hospital, Kaohsiung, Taiwan; 3 Faculty of Renal Care, Kaohsiung Medical University, Kaohsiung, Taiwan; 4 Department of Internal Medicine, Kaohsiung Municipal Hsiao-Kang Hospital, Kaohsiung, Taiwan; University of Louisville, United States of America

## Abstract

Fluid overload is one of the major presentations in patients with late stage chronic kidney disease (CKD). Diabetes is the leading cause of renal failure, and progression of diabetic nephropathy has been associated with changes in extracellular fluid volume. The aim of the study was to assess the association of fluid overload and diabetes in commencing dialysis and rapid renal function decline (the slope of estimated glomerular filtration rate (eGFR) less than -3 ml/min per 1.73 m^2^/y) in 472 patients with stages 4-5 CKD. Fluid status was determined by bioimpedance spectroscopy method, Body Composition Monitor. The study population was further classified into four groups according to the median of relative hydration status (△HS =fluid overload/extracellular water) and the presence or absence of diabetes. The median level of relative hydration status was 7%. Among all patients, 207(43.9 %) were diabetic. 71 (15.0%) subjects had commencing dialysis, and 187 (39.6%) subjects presented rapid renal function decline during a median 17.3-month follow-up. Patients with fluid overload had a significantly increased risk for commencing dialysis and renal function decline independent of the presence or absence of diabetes. No significantly increased risk for renal progression was found between diabetes and non-diabetes in late CKD without fluid overload. In conclusion, fluid overload has a higher predictive value of an elevated risk for renal progression than diabetes in late CKD.

## Introduction

Chronic kidney disease (CKD) has become a global public health problem. Among many traditional risk factors of renal progression, diabetes is the leading cause of CKD in the developed world, and patients with diabetes and CKD have a greater increased risk of end-stage renal disease, and all-cause and cardiovascular mortality than those without diabetes [1]. Progression in diabetic nephropathy has been associated with changes in extracellular fluid volume, thereby further contributing to fluid overload [2]. 

Fluid overload is one of the major presentations in patients with late CKD. The abnormal fluid status has been correlated with arterial hypertension, left ventricular hypertrophy, and other adverse cardiovascular sequelae [3,4]. Accumulating evidence demonstrates the association between fluid overload and a significantly increased risk for all-cause or cardiovascular mortality in dialysis patients [5-8]. Strict volume control would ameliorate survival of dialysis patients [9]. Additionally, our previous study demonstrated a significantly positive relationship between the severity of real fluid status and increased risk for commencing dialysis and rapid renal function decline in late CKD [10]. Hence, fluid overload is not only the characteristic but also an indicator of rapid renal progression in late CKD.

Since both diabetes and fluid overload are significantly associated with renal progression and diabetes has been closely correlated with fluid overload, there might be a complex interaction between diabetes, fluid overload, and renal progression. Many mechanisms have been reported in the causes of renal function decline in diabetes patients [11]. However, the importance of fluid overload in diabetes and whether fluid overload is associated with renal progression independent of diabetes have both been less well explored. Furthermore, which one is more important in clinical practice in late CKD, diabetes or fluid overload? Therefore, the aim of this study was to ascertain the association of fluid status and diabetes in renal progression in CKD stages 4-5 patients not on dialysis.

## Materials and Methods

### Study Participants

All 612 of CKD stages 4-5 patients were invited to participate in the study from January 2011 to December 2011 at one hospital in Southern Taiwan. All patients had been enrolled in our integrated CKD program for more than 3 months (30.9±27.0 months). CKD was staged according to K/DOQI definitions and the estimated glomerular filtration rate (eGFR) was calculated using the equation of the 4-variable Modification of Diet in Renal Disease (MDRD) Study [12]. Overall, 115 with disabilities, 12 with impaired skin integrity, and 5 with pacemaker implantation were excluded. Four hundred and eighty patients were enrolled and scheduled for a study interview after informed consent. Eight patients were excluded because of requiring commencing dialysis within 30 days after enrollment. Patients were censored at the end of follow-up. The final study population comprised 472 CKD stages 4-5 patients in the subsequent analysis. 

### Ethics Statement

The study protocol was approved by the Institutional Review Board of the Kaohsiung Medical University Hospital (KMUH-IRB-990125). Informed consents were obtained in written form from patients and all clinical investigations were conducted according to the principles expressed in the Declaration of Helsinki. The patients gave consent for the publication of the clinical details.

### Measurement of fluid status

Fluid status was measured once by a bioimpedance spectroscopy method, Body Composition Monitor (BCM, Fresenius Medical Care) at enrollment. Based on the difference of impedance in each tissue, BCM provides the information of extracellular fluid overload, normohydrated lean tissue, and normohydrated adipose tissue in whole body. Normal extracellular water (ECW) and intracellular water (ICW) can be determined for a given weight and body composition. Fluid overload can be calculated from the difference between the normal expected and measured ECW [13]. BCM has been validated intensively against all available gold-standard methods in the general and dialysis populations [14-17]. In the present study, fluid overload value, ECW, ICW and total body water (TBW) were determined from the measured impedance data following the model of Moissl et al [15,18]. Only the parameters of fluid status for which the quality of the measurement was 95% or more were included in the analysis. The relative hydration status (△HS =fluid overload/ECW) was used as an indicator of fluid status [7]. Wabel et al. showed that 15% ΔHS could be used as a cutoff for severe fluid overload and 6.8% as mild fluid overload for patients on hemodialysis [7]. In the normal reference population, the 90^th^ percentile of ΔHS was 7%. Accordingly, when ΔHS was greater than 7%, it was classified as “fluid overload” [19]. In this study, ΔHS of 7% or more was defined as fluid overload.

### Assessment of pulse wave velocity (PWV)

The values of PWV were measured by an ankle brachial index-form device, which automatically and simultaneously measured blood pressures in both arms and ankles using an oscillometric method [20]. After obtaining bilateral PWV values, the higher one was used as representative for each subject. The PWV measurement was done once at the same time of fluid measurement for each patient.

### Data Collection

Demographic and clinical data were obtained from medical records and interviews with patients at enrollment. Diabetes was defined as those with a medical history through chart review. Cardiovascular disease was defined as a history of heart failure, acute or chronic ischemic heart disease, and myocardial infarction. Cerebrovascular disease was defined as a history of cerebral infarction or hemorrhage. Information regarding patient medications including diuretics, calcium channel blockers, β-blocker, and angiotensin converting enzyme inhibitors (ACEI), and angiotensin II receptor blockers (ARB) within 3 months before enrollment was obtained from medical records. The participant was asked to fast for at least 12 hours before blood sample collection for the biochemistry study and protein in urine was measured using an immediate semiquantitative urine protein dipstick test and graded as negative, trace, 1+, 2+, 3+, or 4+. The severity of leg edema was also measured and graded individually on a 4-point system [21]. Blood pressure was recorded as the mean of two consecutive measurements with 5-minute intervals, using one single calibrated device. 

### Commencing dialysis

Commencing dialysis was confirmed by reviewing medical charts or catastrophic illness certificate (issued by the Bureau of National Health Insurance in Taiwan) and defined as requiring commencing hemodialysis or peritoneal dialysis. The timing for commencing dialysis was considered according to the regulations of the Bureau of the National Health Insurance of Taiwan regarding the laboratory data, eGFR, uremic status, and nutritional status [22]. 

### Renal function decline

Patients are followed at nephrologic outpatient clinics at three-month intervals to ascertain the renal and vital status. Every patient received eGFR measurement at least every three month. The decline in kidney function was assessed by the eGFR slope, defined as the regression coefficient between eGFR and time in units of ml/min/1.73 m^2^ per year. All eGFR values available from ascertainment of fluid status to the end of the observation period were included for calculation. At least three eGFR values were required to estimate the eGFR slope. Rapid renal progression was defined as the lowest quartile (the eGFR slope < -3 ml/min/1.73 m^2^ per year, an integer near the cutoff point between the lowest two quartiles of the eGFR slope) [22]. 

### Statistical Analysis

The study population was further classified into four groups according to △HS of 7% and the presence or absence of diabetes. Continuous variables were expressed as mean±SD or median (25^th^, 75^th^ percentile), as appropriate, and categorical variables were expressed as percentages. Skewed distribution continuous variables were log-transformed to attain normal distribution. The significance of differences in continuous variables between groups was tested using the one-way analysis of variance (ANOVA) followed by the post hoc test adjusted with a Bonferroni correction or the Kruskal-Wallis H test, as appropriate. The difference in the distribution of categorical variables was tested using the Chi-square test. Time-to-event survival analysis by Kaplan-Meier survival curve was used to test fluid overload or diabetes as a predictor of the risk of commencing dialysis. Cox regression models were utilized to evaluate the relationship between fluid overload and diabetes in commencing dialysis. Multivariable logistic regression models were also utilized to examine the association of renal function decline with fluid overload and diabetes. Age, gender, medication (diuretics and ACEI/ARB), and all the variables in Table 1 tested by univariate analysis and those variables with P-value less than 0.05, including eGFR, cardiovascular disease, serum hemoglobin, hypoalbuminemia (serum albumin level less than 3.5g/dl), calcium-phosphate product cut at 50mg^2^/dl^2^, proteinuria (urine protein above 1+), and systolic blood pressure cut at 140mmHg were selected in multivariate analysis. Statistical analyses were conducted using SPSS 18.0 for Windows (SPSS Inc., Chicago, Illinois). Statistical significance was set at a two-sided p-value of less than 0.05.

**Table 1:** The clinical characteristics of study subjects.

	Entire Cohort (n=472)	△HS<7% Non-Diabetes (n=157)	△HS≧7% Non-Diabetes (n=108)	△HS<7% Diabetes (n=80)	△HS≧7% Diabetes (n=127)	P-value
Demographic variables						
Age, year	65.4±12.7	62.6±13.9**^*†*^**	70.1±12.9^*^	67.1±10.7	63.6±10.9	<0.001
Sex (male), %	54.4	49.7**^*#*^**	52.8**^*#*^**	55.0^*†^	61.4^*†^	0.3
Smoke, %	20.6	15.9	16.7	30.0	23.6	0.02
Alcohol, %	9.7	13.4	6.5	5.0	11.0	0.4
CKD cause						<0.001
Chronic glomerulonephritis, %	31.1	51.6	36.1	16.3	7.1	
Diabetes, %	35.4	2.5	5.6	63.8	83.5	
Others, %	33.5	45.9	58.3	19.9	9.4	
Cardiovascular disease, %	18.4	10.2**^*#*^**	20.4	27.5^*^	21.3	0.01
Cerebral vascular disease, %	9.5	3.8	12.0	10.0	14.2^*^	0.02
CKD stage 4, %	49.8	51.6	46.3	55.0	47.2	0.6
5, %	50.2	48.4	53.7	45.0	52.8	
Body Mass Index, kg/m^2^	24.3±3.8	23.7±3.3	23.0±3.1	26.5±3.9	24.9±4.1	<0.001
Systolic blood pressure, mmHg	139.6±20.3	131.0±16.3**^*†#*^**	140.4±20.8^*^	139.0±18.0^*^	150.1±21.0^*†^^*#*^	<0.001
Diastolic blood pressure, mmHg	75.6±11.6	76.7±10.8	74.1±12.7	75.6±10.8	75.6±11.9	0.4
Leg edema severity≧1, %	18.4	3.8**^*†*^**	24.6^*^	7.4	40.0^*#^	<0.001
Pulse wave velocity (per 100cm/s)	18.6±3.9	17.0±3.2**^*†#*^**	19.1±3.9^*^	19.3±4.0^*^	19.9±4.1^*^	<0.001
Body Composition						
Lean tissue Index (kg/m^2^)	13.7±2.6	14.1±2.6**^*†*^**	13.0±2.4^*^	14.0±2.7	13.7±2.8	0.01
Fat tissue Index (kg/m^2^)	9.8±4.2	9.3±4.1**^*#*^**	8.9±3.5**^*#*^**	12.3±4.6^*†^	9.7±4.2**^*#*^**	<0.001
Total body water (L)	32.7±6.7	32.0±5.9	31.2±6.2	32.9±6.2	34.7±7.8^*†^	<0.001
Intracellular water (L)	17.1±3.6	17.5±3.5**^*†*^**	16.0±3.4^*#^	17.5±3.2**^*†*^**	17.3±4.0**^*†*^**	0.004
Extracellular water (L)	15.6±3.6	14.5±2.8	15.2±2.9	15.4±3.7	17.4±4.1^*†^^*#*^	<0.001
ECW/ICW	0.9±0.1	0.8±0.1**^*†#*^**	1.0±0.1^*#^	0.9±0.2	1.0±0.1^*†^^*#*^	<0.001
ECW/TBW (%)	47.7±3.5	45.4±2.9**^*†#*^**	48.9±2.3^*#^	46.7±3.2^*†^	50.2±3.0^*†^^*#*^	<0.001
Fluid overload (L)	0.9 (0.4,2.2)	0.4(-0.1,0.7)**^*†*^**	1.7(1.3,2.4)^*#^	0.5(-0.1,0.8)**^*†*^**	2.7(1.8,4.1)^*†^^*#*^	<0.001
Medications						
Diuretics, %	29.0	12.1**^*#*^**	22.2	35.0^*^	52.0^*†^^*#*^	<0.001
Calcium channel blocker, %	59.7	40.1**^*†#*^**	59.3^*^	66.3^*^	80.3^*†^	<0.001
β-blocker, %	27.8	15.9**^*#*^**	26.9	35.0^*^	38.6^*^	<0.001
ACEI/ARB, %	53.0	45.2**^*#*^**	41.7**^*#*^**	68.8^*†^	62.2^*†^	<0.001
Laboratory parameters						
Blood urea nitrogen, mg/dl	47.0(34.1,63.8)	41.9(31.7,62.8)	48.2(35.5,63.8)	42.5(34.0,59.7)	55.1(39.4,68.8)^*^	0.002
eGFR, ml/min/1.73m2	15.4 ± 7.5	15.9 ± 7.8	14.5 ± 6.9	16.8 ± 8.1	14.8 ± 7.0	0.1
Glycated hemoglobin, %	5.9(5.6, 6.8)	5.6(5.5,5.9)**^*#*^**	5.6(5.4,5.8)**^*#*^**	6.6(6.0,7.7)^*†^	6.8(6.0,7.6)^*†^	<0.001
Hemoglobin, g/dl	10.4 ± 1.7	10.7 ± 1.8**^*†*^**	9.9 ± 1.6^*#^	10.9 ± 1.5**^*†*^**	10.2 ± 1.8	<0.001
Albumin, g/dl	4.0 ± 0.4	4.3 ± 0.3**^*†*^**	4.0 ± 0.4^*#^	4.1 ± 0.3**^*†*^**	3.8 ± 0.5^*†^^*#*^	<0.001
Calcium and Phosphate product	38.3(34.2, 43.9)	37.7(33.4,43.4)	38.5(33.7,43.4)	39.6(33.0,46.3)	38.1(35.2,44.1)	0.6
Uric acid, mg/dl	7.7 ± 1.7	7.3 ± 1.6	7.6 ± 1.8	7.8 ± 1.7	8.1 ± 1.7^*^	<0.001
Cholesterol, mg/dl	180(153, 210)	185(161,212)	170(147,206)	183(163,210)	179(143,206)	0.07
Triglyceride, mg/dl	116(80, 165)	107(82,159)**^*#*^**	89(66,142)**^*#*^**	161(99,229)^*†^	125(85,179)**^*†*^**	<0.001
hsCRP, mg/L	1.3(0.6, 3.6)	1.2(0.6,2.2)	1.2(0.6,3.6)	2.0(1.0,4.4)	1.3(0.5,4.0)	0.07
Urine protein above 1+^a^ (%)	49.0	28.9**^*†*^**	46.0^*^	46.3	78.7^*†^^*#*^	<0.001
Parathyroid hormone, pg/ml	93(50, 234)	86(44,227)	126(60,273)	71(44,187)	98(50,226)	0.2

Notes: Data are expressed as number (percentage) for categorical variables and mean±SD or median (25^th^, 75^th^ percentile) for continuous variables, as appropriate.

Conversion factors for units: eGFR in mL/min/1.73 m^2^ to mL/s/1.73 m^2^, ×0.01667 ; hemoglobin in g/dL to g/L, ×10; albumin in g/dL to g/L, ×10; calcium in mg/dL to mmol/L, ×0.2495; phosphate in mg/dL to mmol/L, ×0.3229; cholesterol in mg/dL to mmol/L, ×0.02586; triglyceride in mg/dL to mmol/L, ×0.01129; uric acid in mg/dL toμmol/L, ×59.48

Abbreviations: CKD, chronic kidney disease; △HS, relative hydration status, ECW, extracellular water; ICW, intracellular water; TBW, total body water; ACEI, angiotensin converting enzyme inhibitors; ARB, angiotensin II receptor blockers; eGFR, estimated glomerular filtration rate; hsCRP, high-sensitivity C-reactive protein

^a^ Urine protein was measured using dipstick test.

^*^ *P* < 0.05 compared with △HS<7% and non-diabetes; ***^†^*** *P* < 0.05 compared with △HS≧7% and non-diabetes; ***^#^*** *P* < 0.05 compared with △HS<7% and diabetes.

## Results

### Characteristics of Entire Cohort

The median levels of fluid overload and fluid overload/ECW (△HS) of the entire cohort were 0.9 L and 7%, respectively. The comparison of clinical characteristics between groups based on △HS divided at 7% and the presence or absence of diabetes is shown in Table 1. The mean age was 65.4±12.7 years. A total of 257 (54.4%) and 207 (43.9%) were male and had diabetes, respectively. The patients with both fluid overload and diabetes had the highest systolic blood pressure (150.1±21.0mmHg) and highest prevalence of cerebral vascular disease (14.2%) than other patients. Diabetic patients without fluid overload had higher percentage of cardiovascular disease (27.5%) and the highest body mass index (26.5±3.9 kg/m^2^) among all patients. Diabetic patients with fluid overload received more diuretics, calcium channel blocker, and β-blocker treatment. TBW, ECW and ECW/TBW were higher in patients with both diabetes and fluid overload than those with only diabetes or fluid overload. Blood urea nitrogen, serum glycated hemoglobin and uric acid levels, and the severity of urine protein were the highest and serum albumin was the lowest in patients with diabetes and fluid overload than others. 

### Fluid overload and commencing dialysis

**Figure 1:**
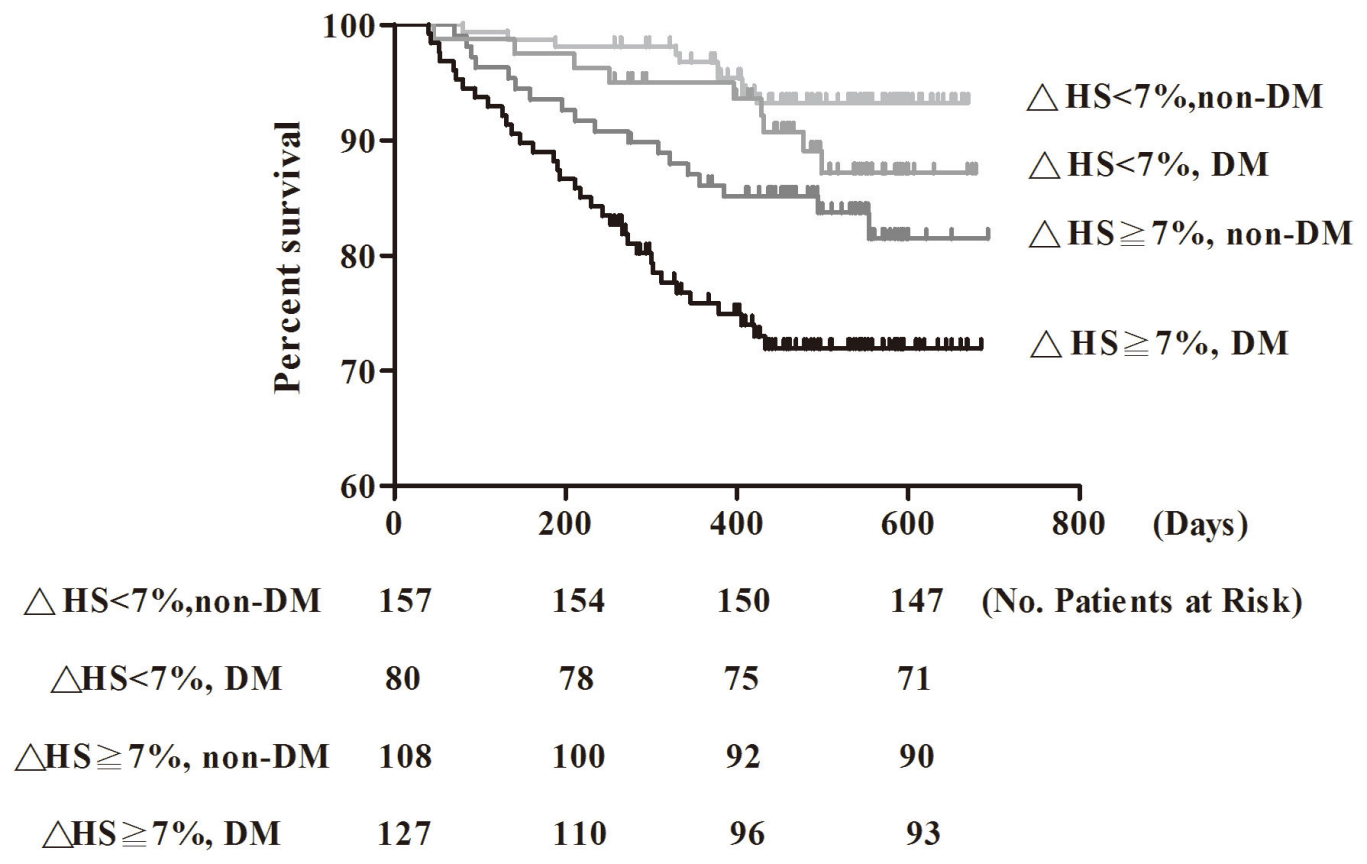
Kaplan-Meier survival curve for commencing dialysis based on fluid overload (relative hydration status, △HS≧7%) and diabetes mellitus (DM).

Seventy-one patients (15.0%) progressed to commencing dialysis, 65 of hemodialysis and 6 of peritoneal dialysis during a median follow-up of 17.3 (14.0, 19.1) months (Table 2). No more fatal events were recorded. Patients with both diabetes and fluid overload were more likely to reach commencing dialysis compared to others (26.8%). As documented by the Kaplan-Meier curves (Figure 1), fluid overload was significantly associated with commencing dialysis in the presence or absence of diabetes. We further analyzed the association between fluid overload and diabetes in commencing dialysis using cox regression analysis (Table 3). △HS was significantly associated with high risk for commencing dialysis 〔every 1 unit increase hazard ratio (HR): 1.07, 95% Confidence Interval (CI):1.03-1.11), P=0.001〕. The unadjusted and adjusted HR for commencing dialysis in non-diabetic patients with fluid overload compared with those without fluid overload was 2.73(95% CI: 1.26-5.92, P=0.01)and 2.96 (95% CI: 1.13-7.72, P=0.03) respectively. The unadjusted and adjusted HR for commencing dialysis in diabetic patients with fluid overload compared with non-diabetic patients without fluid overload was 5.04 (95% CI: 2.49-10.21, P-value<0.001) and 4.48 (95% CI: 1.69-11.93, P=0.003) respectively. A significant interaction between diabetes and fluid overload was shown in multivariate analysis of progression of commencing dialysis (HR: 1.48, 95% CI: 1.03-2.11, P=0.03). However, no significantly increased risk for commencing dialysis was shown between diabetes and non-diabetes in late CKD without fluid overload. 

**Table 2:** Renal progression of all subjects.

	Entire Cohort (n=472)	△HS<7% non-Diabetes (n=157)	△HS≧7% non-Diabetes (n=108)	△HS<7% Diabetes (n=80)	△HS≧7% Diabetes (n=127)	P-value
Follow-up time (month)	17.3(14.0, 19.1)	17.9(14.9,19.4)	17.2(14.9,19.1)	17.9(15.0,19.4)	15.3(9.6, 18.6)	0.001
No. of SCr measurement	7 (4,10)	6(4,11)	7(5,11)	7(4,10)	7(4,10)	0.8
eGFR decline (mL/min/1.73 m^2^ /year)	-2.0(-5.3, -0.1)	-1.2(-2.5,0.5)**^*†*^**	-3.3(-5.9,-0.9)^*#^	-1.2(-3.1,1.4)**^*†*^**	-3.7(-7.9,-0.6)^*#^	0.003
Dialysis, n(%)	71(15.0)	10(6.4)	18(16.7)	9(11.3)	34(26.8)	<0.001

Notes: Data are expressed as number (percentage) for categorical variables and median (25^th^, 75^th^ percentile) for continuous variables, as appropriate.

Conversion factors for units: eGFR in mL/min/1.73 m^2^ to mL/s/1.73 m^2^, ×0.01667

Abbreviations: CKD, chronic kidney disease; △HS, relative hydration status

^*^ *P* < 0.05 compared with △HS<7% and non-diabetes; ***^†^*** *P* < 0.05 compared with △HS≧7% and non-diabetes; ***^#^*** *P* < 0.05 compared with △HS<7% and diabetes

**Table 3:** The risks for commencing dialysis and renal function progression.

	Commencing dialysis HR(95% CI)				Renal function progression OR(95% CI)			
	Unadjusted Model	P-value	Adjusted Model	P-value	Unadjusted Model	P-value	Adjusted Model	P-value
△HS<7%,non-DM	1(Reference)		1(Reference)		1(Reference)		1(Reference)	
△HS≧7%, non-DM	2.73(1.26-5.92)	0.01	2.96(1.13-7.72)	0.03	3.00(1.78-5.05)	<0.001	4.26(2.08-8.73)	<0.001
△HS<7%, DM	1.81(0.74-4.45)	0.2	1.94(0.69-5.46)	0.2	1.42(0.78-2.56)	0.2	0.66(0.28-1.58)	0.3
△HS≧7%, DM	5.04(2.49-10.21)	<0.001	4.48(1.69-11.93)	0.003	2.74(1.66-4.51)	<0.001	2.82(1.27-6.26)	0.01

Abbreviations: HR, Hazard ratio; OR, Odds Ratio; CI, Confidence Interval; △HS, relative hydration status; DM, diabetes

Adjusted model: age, sex, cardiovascular disease, medication including angiotensin converting enzyme inhibitors/angiotensin II receptor blockers and diuretics, estimated glomerular filtration rate, hemoglobin level, hypoalbuminemia (serum albumin level less than 3.5g/dl), calcium-phosphate product cut at 50mg^2^/dl^2^, proteinuria (urine protein above 1+), systolic blood pressure cut at 140mmHg

### Fluid overload and renal function decline

No significant difference of the number of eGFR measurement among four groups was found. The median interval of two eGFR measurements was less than two months and at least 4 data of serum creatinine for slope calculation for every patient. Table 2 showed that the decline in renal function in patients with fluid overload (eGFR slope: -3.7(-7.9,-0.6) and -3.3(-5.9,-0.9) mL/min/1.73 m^2^/year respectively) was significantly faster than those without fluid overload (eGFR slope: -1.2(-3.1,1.4) and -1.2(-2.5,0.5) mL/min/1.73 m^2^/year respectively) independent of diabetes. △HS was significantly associated with high risk for renal function decline 〔every 1 unit increase odds ratio (OR): 1.06, 95% CI:1.02-1.10), P=0.001〕. The unadjusted and adjusted odds ratio (OR) for renal function decline in non-diabetic patients with fluid overload compared with those without fluid overload was 3.00 (95% CI: 1.78-5.05, P-value<0.001) and 4.26 (95% CI: 2.08-8.73, P<0.001, Table 3) respectively. The unadjusted and adjusted OR for renal function decline in diabetic patients with fluid overload compared with non-diabetic patients without fluid overload was 2.74 (95% CI: 1.66-4.51, P-value<0.001) and 2.82 (95% CI: 1.27-6.26, P=0.01) respectively. Nevertheless, no significantly increased risk for renal function decline was found between diabetes and non-diabetes in stages 4-5 CKD without fluid overload. 

## Discussion

This study is the first to analyze the separate effect of fluid overload and diabetes on commencing dialysis or renal function decline in patients with stages 4-5 CKD. Fluid overload combined with diabetes together have an additive interaction of an increased risk for commencing dialysis. Patients with fluid overload are more likely to have a significantly increased risk for commencing dialysis and renal function decline independent of diabetes. The survival of renal progression between diabetes and non-diabetes is not significantly different in late CKD cohort without fluid overload.

The association between CKD progression and fluid overload may be confounded by several traditional risk factors for renal function decline [23,24]. Among these factors, diabetes is the most common cause of progression to renal failure, with nearly 40% of patients with CKD affected by diabetes [25]. Accumulating evidence demonstrates that strict glycemic control shows great benefit in preventing the development of microalbuminuria and overt nephropathy [26,27]. However, Shurraw et al. demonstrated that the association between better glycemic control and decreased risk for commencing dialysis was attenuated in late CKD. This finding indicated an issue whereby intensive glycemic control may not be enough to prevent progressive renal function decline in late CKD [28]. There could be some causes which have a stronger influence on renal progression than diabetes in late CKD. Our results present that fluid overload is associated with renal progression independent of the presence or absence of diabetes in late CKD. No significant difference of cox proportional hazard is found in late CKD without fluid overload between diabetes and non-diabetes. Besides, the present results also show an additional effect between diabetes and fluid overload on renal progression. Therefore, fluid overload seems to have a stronger influence on renal progression than diabetes in late CKD. However, the small number of late CKD without fluid overload and short observation period are needed to be considered for this finding. Large study is needed for evaluate the role of diabetes on renal progression in late CKD.

High blood sugar stimulates the patient’s thirst and increases dietary fluid intake and the patients become more hydrated. The imbalance of renin-angiotensin aldosterone system (RAAS) in diabetic nephropathy was also associated with changes in extracellular fluid volume [29]. Increased capillary permeability caused by both diacylglycerol and advanced glycosylation end products (AGEs) [30,31] may aggravate the excess volume status in the interstitium and cause erroneous judgments about intra-vascular fluid status. These effects may lead to fluid overload in diabetes patients. However, what is the role of fluid overload in renal function deterioration that leads to dialysis in these diabetes patients? The present study demonstrates that fluid overload was independently associated with commencing dialysis and renal function decline in the presence or absence of diabetes. These findings show the severity of fluid overload as a predictor of renal progression and emphasize its importance as a predictor in the presence or absence of diabetes. 

Though diabetic patients have usually been associated with fluid overload [32], a substantial proportion of patients did not comply with this paradigm in the present study. Some patients had diabetes, despite normohydration or even tissue underhydration. These are probably patients who suffer from arterial stiffness. Increased arterial stiffness results in greater transmission of elevated systemic blood pressure to the glomerular capillaries, thereby exacerbating glomerular hypertension, a major determinant of progressive renal damage [33]. Our results show that both diabetes and fluid overload are associated with higher pulse wave velocity, as a marker of arterial stiffness in the present study (data not shown). In addition to arterial stiffness, some factors, such as malnutrition and inflammation may contribute to renal function decline through fluid overload. This may partially explain that those non-diabetic patients with higherΔHS have higher risk for commencing dialysis and renal function decline than diabetic patients with lowerΔHS. Further study is needed to evaluate the mechanism between fluid overload and renal progression. 

Evaluation of volume status is an important issue in CKD patients in clinical practice. Essig et al. indicated that only 8% increase in ECW might easily be underestimated by clinical examination [23]. Physical examination is not enough to detect small increases in volume status efficiently. ECW/TBW is frequently expressed as actual volumes, but the ratio is often affected by muscle wasting and abnormal tissue hydration. The relative hydration status (△HS =fluid overload/ECW) has been regarded as an indicator of fluid status [7] Wabel et al. showed that 15% ΔHS could be used as a cutoff of severe fluid overload and a predictor of adverse outcome in dialysis patients [7]. The present study reveals that CKD patients with △HS of 7% or more had a higher risk for renal progression. These findings show CKD patients may have lower threshold of influence of fluid overload than patients on dialysis. Hence, objective and quantitative assessment of fluid status and combining important clinical information might be the first step to accomplish further advancements in relieving renal function decline. 

This study has some limitations that must be mentioned. Fluid status and the other clinical variables were measured only once at enrollment. The effect of time-varying fluid status and the other clinical variables on renal progression might be underestimated. The BCM has been validated in the dialysis population, but not in CKD populations. This is indeed a limitation of the reality of the parameters of fluid status measured by BCM in CKD populations. Besides, we recorded use of ACEI/ARB and diuretics as categorical variables within 3 months before enrollment, not time-averaged defined daily dose of these drugs. The effect of time-varying dose of medication might be underestimated. 

In conclusion, our study demonstrates that fluid overload leads to an increased risk for commencing dialysis and renal function decline in stages 4-5 CKD patients in the presence or absence of diabetes. Fluid overload has a higher predictive value of an elevated risk for renal progression than diabetes in late CKD. Future intervention studies will be necessary to demonstrate whether objective identification and relief of fluid overload can slow down the renal function decline in CKD patients with and without diabetes. 
